# 640. A County-wide Collaboration to Drive Quality Improvement Using Healthcare-associated Infection Surveillance Data from the National Healthcare Safety Network, Centers for Disease Control and Prevention

**DOI:** 10.1093/ofid/ofae631.205

**Published:** 2025-01-29

**Authors:** Madeleine Monroe, Grace Kang, Francesca J Torriani, Frank Myers, Karen Meyer, Priscila de Vera, Jarrod Becasen, Shannon C Mabalot, Kerry Schultz, Peter binstock, Wun-Ling Chang, Stacie Benedek, Tamara Hayes, elizabeth A jefferson, Shweta Warner, Tamara Behm, Sandeep Soni, Kyle Mataya, Erik A Berg, Aaron Menz, Alexis Nickel, Mary Grace Sadile, Annie S Kao, Jeffrey Johnson, Heidi Aiem, Mark E Beatty, Seema Shah, Raymond Chinn

**Affiliations:** County of San Diego Health and Human Services Agency, San Diego, California; County of San Diego, Epidemiology & Immunization Services Branch, San Diego, California; University of California, San Diego School of Medicine, San Diego, California; University of California San Diego, San Diego, California; Scripps Health, San Die, California; Kaiser Permanente, San Diego, California; Palomar Health, Escondido, California; Sharp Memorial Hospital, San Diego, CA; Sharp Mary Birch Hospital for Women and Newborns, SAN DIEGO, California; Sharp Metro Campus, San Diego, California; Scripps Memorial La Jolla Hospital, San Diego, California; Scripps Health, San Die, California; Scripps Mercy Hospital, San Diego, California; Scripps Green Hospital, la jolla, California; Kaiser Permanente San Diego, San Diego, California; Kaiser Permanent San Diego, san diego, California; Palomar Health, Escondido, California; paradise valley hospital, San Diego, California; County of San Diego, San Diego, California; County of San Diego, San Diego, California; County of San Diego - Public Health Services, San Diego, California; County of San Diego, San Diego, California; County of San Diego, HHSA, San Diego, California; County of San Diego Health and Human Services Agency, San Diego, California; County of San Diego, Health & Human Services Agency: Public Health Services, San Diego, California; County of San Diego Health and Human Services Agency, San Diego, California; County of San Diego, Health and Human Services Agency, San Diego, CA; County of San Diego, Health and Human Services Agency, San Diego, CA

## Abstract

**Background:**

Quality healthcare is dependent on continuous input and feedback. The California Department of Public Health (CDPH) requires that all general acute care hospital (GACH) report healthcare-associated infections (HAI) annually. Comparisons of current facility-specific standardized infection ratios (SIRs) to the National Healthcare Safety Network (NHSN) 2015 SIR baseline provide designations of whether a facility is statistically significantly better, worse, or average are displayed for public reporting purposes. Since then, the annual NHSN and CDPH SIR baselines have dropped below 1 for many HAIs; however, neither NHSN nor CDPH provide facility-specific comparisons to the most recent annual NHSN and CDPH SIRs. Facilities that are performing better or average using the 2015 baseline may be performing average or worse when compared the the most recent baselines (Figure A.).

**Figure:**
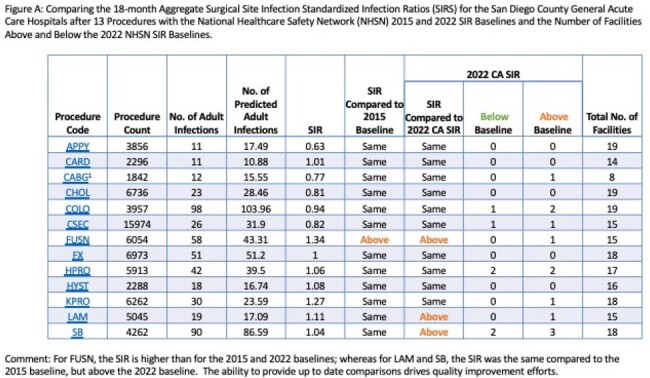

**Methods:**

The County of San Diego (COSD) HAI program began a collaborative effort with local GACHs in 2020 (Figure B). A work group comprised of Infection Preventionists and Epidemiologists convened to provide input into the format and content. Unique features include: 1) a display of an 18 month select HAI aggregate SIRS for the GACHs; 2) statistical analysis using a NHSN SAS 9.4 Macro to provide facility-specific comparative SIRs to the most recent annual NHSN and CDPH baselines and an 18-month aggregate COSD SIR baselines. Facilities were de-identified; the data were distributed only to Infection Prevention Departments for quality improvement and not used for regulatory purposes,; and met the intent of the NHSN Data Use Agreement.

**Figure:**
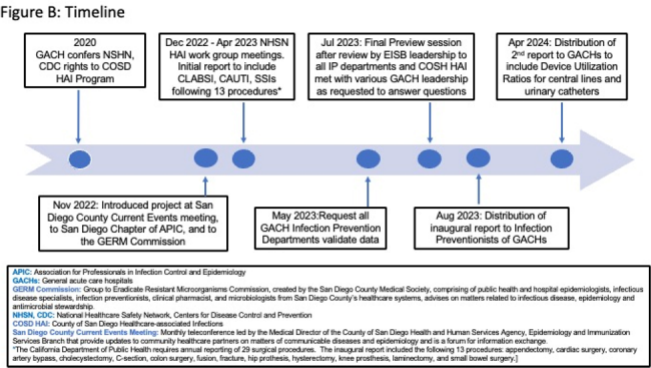

**Results:**

The first report (8/2023) included comparative SIRs on13 surgeries and central-line associated blood stream and catheter-associated urinary tract infections. The second report (4/2024) adds comparative standardized utilization ratios for central lines and urinary catheters (Figures C, D).

**Figure:**
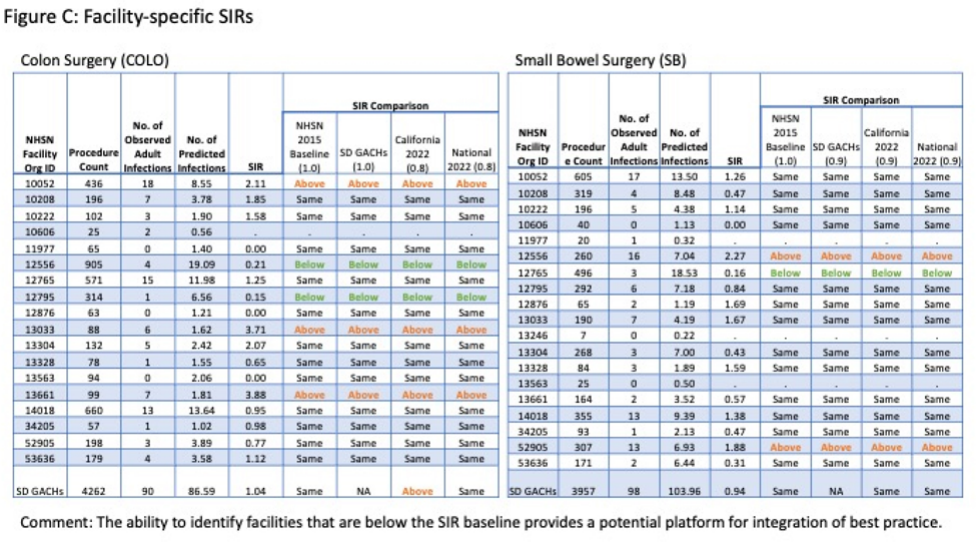

**Conclusion:**

Through this effort, the COSD HAI program has strengthened communication between our local health department and the GACHs and also between GACHs. Future reports will be refined based on the input from the GACHs and could include comparative surveillance data on other HAIs being reported to NHSN and CDPH. Sharing best practices could result in continued decreases in HAIs.

**Figure:**
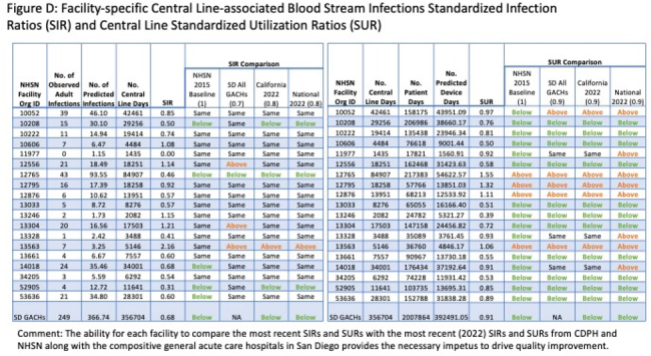

**Disclosures:**

**All Authors**: No reported disclosures

